# Hot-Melt Pneumatic Extrusion-Based 3D-Printed Bilayer Tablets Enabling Sequential Release of Levocetirizine and Montelukast

**DOI:** 10.3390/pharmaceutics18040444

**Published:** 2026-04-03

**Authors:** Ga-Ram Kim, Ji-Young Cho, Seung-Wuk Lee, Hyo-Eon Jin

**Affiliations:** 1 Department of Biohealth Regulatory Science, Graduate School of Ajou University, Suwon 16499, Republic of Korea; garam0431@ajou.ac.kr (G.-R.K.); jyoung919@ajou.ac.kr (J.-Y.C.); 2 Department of Bioengineering, University of California, Berkeley, CA 94720, USA; leesw@berkeley.edu; 3 Department of Pharmacy, Ajou University, Suwon 16499, Republic of Korea

**Keywords:** three-dimensional printing technology, bilayer tablet, sequential release, levocetirizine dihydrochloride, montelukast sodium

## Abstract

**Background/Objectives**: This study aimed to develop bilayer tablets using hot-melt pneumatic extrusion (HMPE)-based 3D printing for the integrated treatment of allergic rhinitis and asthma. The formulation combined levocetirizine dihydrochloride (immediate release) and montelukast sodium (delayed release) within a single dosage form to provide a sequential-release formulation strategy relevant to the intended pharmacological roles of the two drugs. Distinct polymer matrices were selected for each drug layer to ensure mechanical robustness, stability, and appropriate release characteristics. **Methods**: The printed tablets were systematically characterized by mechanical testing, differential scanning calorimetry (DSC), powder X-ray diffraction (PXRD), and in vitro dissolution. Drug content uniformity was evaluated in accordance with USP <905>. **Results**: The tablets satisfied USP standards for content uniformity and exhibited sufficient mechanical strength for handling and packaging. DSC and PXRD analyses indicated amorphization of levocetirizine within the polymer matrix, while the amorphous state of the raw montelukast used in this study was retained after printing. In vitro dissolution tests demonstrated immediate release of levocetirizine in acidic medium (pH 1.2) and delayed release of montelukast at intestinal pH (6.8), thereby achieving the intended dual-phase release profile. **Conclusions**: These findings demonstrate the feasibility of fabricating an HMPE-based 3D-printed bilayer tablet integrating immediate-release levocetirizine and delayed-release montelukast, with reproducible dual-phase release and drug-specific solid-state and performance characteristics within a single oral dosage form.

## 1. Introduction

Three-dimensional (3D) printing has emerged as a versatile platform for precise drug delivery and the rapid development of complex dosage forms, enabling dose individualization and geometry-guided release directly from digital designs [1,2,3]. Compared with conventional multi-step manufacturing, extrusion-based 3D printing can reduce process complexity by fabricating units after simple blending of active pharmaceutical ingredients (APIs) with excipients [4,5,6]. Among 3D printing techniques, hot-melt pneumatic extrusion (HMPE) is a filament-free, direct powder extrusion approach that enables single-step printing of multi-compartment constructs, broadens formulation space, and limits repeated thermal exposure relative to fused deposition modelling (FDM), which typically requires prior hot-melt extrusion of drug-loaded filaments [7,8]. Importantly, HMPE can also improve the bioavailability of drugs with limited solubility or gastric instability by enabling optimized dispersion and controlled release profiles [7,9,10]. These capabilities are advantageous for APIs requiring amorphous dispersion, gastric protection, or tailored release [11].

Allergic rhinitis is highly prevalent and frequently coexists with asthma, contributing to substantial symptom burden and impaired quality of life [12,13]. Growing evidence supports the concept that upper- and lower-airway inflammation form a single respiratory continuum (‘one airway’), motivating coordinated management strategies [14,15]. Histamine and leukotrienes are key mediators in allergic rhinitis pathophysiology. Levocetirizine rapidly attenuates histamine-driven early-phase symptoms (itching, sneezing, rhinorrhea) [16,17], whereas montelukast targets leukotriene-mediated late-phase and nocturnal components, including mucosal edema and airflow limitation [18,19]. Co-locating these actions within a single bilayer tablet containing an immediate release (IR) layer and delayed release (DR) layer operationalizes pathway-complementarity without increasing pill burden, consistent with randomized and pooled evidence indicating complementary day- and night-time symptom control compared with monotherapy [20,21,22]. Accordingly, second-generation H1-antihistamines and leukotriene receptor antagonists (LTRAs) are core pharmacotherapies, and co-administration has been reported to improve overall disease control and may support treatment adherence [23,24,25].

Chronotherapeutic and biopharmaceutical considerations suggest staggered onset within a single dosage form. Montelukast sodium, a widely used LTRA, is typically given in the evening to address nocturnal symptom exacerbation [21]. However, evening dosing is vulnerable to missed doses, neuropsychiatric adverse events may be under-recognized, and montelukast’s gastric-labile nature can reduce efficacy and increase gastrointestinal irritation [26,27]. A DR design that releases montelukast in the intestine approximately two hours after administration may provide a formulation strategy relevant to its intended use during nocturnal symptom exacerbation while minimizing gastric exposure [28]. By contrast, levocetirizine requires a rapid onset (~1 h) and sustained peripheral H1-blockade, making an IR appropriate to control acute histamine-mediated symptoms [29].

Within this ‘one airway’ framework, a single bilayer tablet combining IR levocetirizine and DR montelukast is designed to coordinate complementary histamine- and leukotriene-mediated pathways while reflecting chrono-pharmacological considerations at the formulation design level. HMPE-based 3D printing offers a filament-free, single-step process that enables precise control of layer thickness and spatial drug distribution without prior filament optimization, while limiting repeated thermal exposure—an advantage for acid-labile or thermosensitive APIs [7,9,10]. In addition, the administered dose in HMPE-based 3D printing is intrinsically linked to the printed mass, allowing accurate and reproducible drug loading without reliance on powder flow, die filling, or compression behavior typical of conventional tableting [30]. The digitally defined, single-step process also eliminates the high shear stress and torque associated with screw-based hot-melt extrusion, thereby reducing mechanical and thermal stress on APIs. Once printing parameters are fixed, this digitally driven platform offers high batch-to-batch reproducibility, which is particularly advantageous for multilayered dosage forms requiring precise control of layer geometry and release performance [1,31].

Unlike conventionally compressed bilayer tablets, which are limited to fixed dose ratios and predefined release characteristics, HMPE-based 3D printing enables digital control over layer geometry, drug distribution, and release timing. This flexibility provides a platform for programmable sequential release and potential patient-specific combination therapy, which are difficult to achieve using traditional tableting processes.

In this study, we developed a bilayer tablet using HMPE-based 3D printing that integrates an immediate-release levocetirizine layer with a delayed-release montelukast layer. The formulations were optimized to achieve distinct release behaviors for each drug, and the printed tablets were systematically evaluated in terms of mechanical properties, content uniformity, solid-state characteristics, and in vitro dissolution performance. The novelty of the present study lies not simply in the fabrication of a bilayer tablet itself, but in the use of a filament-free HMPE-based 3D printing strategy to produce a bilayer dosage form combining levocetirizine and montelukast for sequential release within a single oral dosage form. Unlike conventional bilayer tablets, which often require additional coating processes or multiple manufacturing steps to achieve delayed-release characteristics, the present system enables spatially programmed drug release through a single-step HMPE printing process. This approach allows precise control over layer geometry, spatial drug distribution, and release timing, thereby providing a formulation platform for sequential combination therapy. Through this approach, we aimed to demonstrate the capability of HMPE-based 3D printing as a platform for sequential combination therapy within a single oral dosage form.

## 2. Materials and Methods

### 2.1. Materials

Montelukast sodium (purity ≥ 98.0%) was obtained from Angene Chemical (London, UK), and levocetirizine dihydrochloride (purity ≥ 98.0%) was purchased from BLD Pharmatech Ltd. (Shanghai, China). Soluplus^®^ (BASF Pharma, Florham Park, NJ, USA), Kollidon^®^ VA 64 (PVPVA64, BASF Pharma, Florham Park, NJ, USA), POLYOX WSR N10 LEO (PEO, Colorcon Ltd., Dartford, UK), hypromellose acetate succinate, MG grade (Shin-Etsu Chemical Co., Ltd., Tokyo, Japan), and Kolliphor^®^ P 188 (poloxamer 188, BASF Pharma, Florham Park, NJ, USA) were utilized as excipients in tablet formulations. Monterizine Cap.^®^, which was used as the reference drug, was obtained from Hanmi Pharm Co., Ltd. (Seoul, Republic of Korea) and is supplied as a tablet-in-capsule dosage form. All other chemicals used in this study were of analytical grade.

### 2.2. Design of 3D-Printed Tablets

Bilayer tablet design was created using Autodesk Fusion 360 software version 2606.1.36 (Autodesk, Inc., San Rafael, CA, USA). Slicer settings were configured via NewCreatorK software version 1.57.71.0 (ROKIT INVIVO Corp., Seoul, Republic of Korea) to ensure compatibility with a HMPE 3D printer (ROKIT INVIVO Corp., Seoul, Republic of Korea), following parameters previously described by Cho et al. [7]. The model files were converted to G-code using NewCreatorK software.

**Figure 1:**
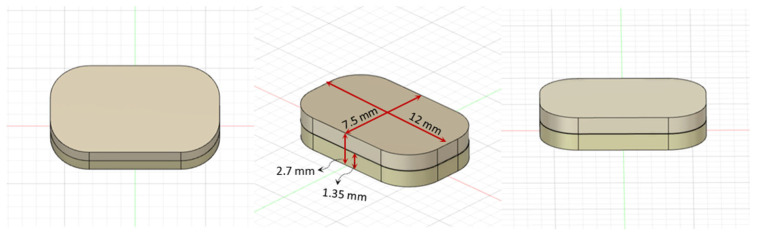
Multi-angle views of the 3D model showing its geometric and structural features. The tablet is designed with a thickness of 12 mm × 7.5 mm × 1.35 mm for each layer.

The tablet was designed in the software such that each layer measured 12 × 7.5 × 1.35 mm (width × length × height), resulting in a total tablet dimension of approximately 12 × 7.5 × 2.7 mm (Figure 1). The tablet dimensions were designed to correspond to the final bilayer tablet containing 10.4 mg of montelukast sodium (MLS) and 5 mg of levocetirizine dihydrochloride (LCD).

### 2.3. Preparation of HMPE

Various ratios of polymers and APIs were weighed and mixed uniformly using a mortar and pestle for 10 min. For process optimization, various ratios of polymer mixtures without APIs were first tested under different extrusion temperatures and air pressure. Each mixture was prepared with a final weight of 3 g, filled into the extrusion barrel, and melted before extrusion. During this optimization step, the temperature was initially set to 80 °C and then increased in 10 °C intervals every 30 min. Once the polymer matrix melted, the temperature was further increased by 2–3 °C to achieve proper flow. Tablets were printed using a HMPE 3D printer equipped with a 0.3 mm nozzle. The printing bed temperature varied depending on the polymer mixture and was optimized accordingly. Air pressure was initially set at 100 kPa and increased in 50 kPa increments. Stable extrusion was achieved at air pressures of 200 kPa and 500 kPa across all formulations. After optimizing all printing conditions, the API-containing mixtures were prepared and used for 3D printing under the selected final processing conditions. Because the two layers were printed sequentially and the barrel temperature was increased stepwise before deposition, the approximately 1 h required for fabrication represented the overall printing process for one bilayer tablet rather than the continuous residence time of each formulation at the final set temperature in the heated barrel. Therefore, the actual exposure time of each formulation at 100 °C (MLS) or 127 °C (LCD) was shorter than the total fabrication time.

### 2.4. Evaluation of the Mechanical Properties of Tablets

The dimensions of the tablets were measured using a vernier caliper (Mitutoyo, Kanagawa, Japan). Tablet hardness was measured using a digital force gauge (HF-500, M&A Instruments Inc., Arcadia, CA, USA). Tablets were placed between two flat plates, and a diametral compressive force was applied at a constant loading rate until fracture occurred. The maximum force required to break the tablet was recorded as the crushing strength. Friability of the printed bilayer tablets was evaluated using a Roche friabilator according to USP guidelines. Ten tablets were weighed collectively, placed in the friabilator, and rotated at 25 rpm for 4 min (100 revolutions). After testing, the tablets were dedusted and reweighed. Friability was calculated as the percentage weight loss relative to the initial total tablet weight.

### 2.5. Ultra High-Performance Liquid Chromatography (UHPLC) Analysis

UHPLC analysis for the quantitative determination of LCD and MLS was performed using a 1290 Infinity II UHPLC system (Agilent Technologies, Santa Clara, CA, USA) equipped with a diode array detector (DAD) set at 220 nm. Separation was performed on a Kinetex^®^ 2.6 μm XB-C18 LC column (100 × 2.1 mm) (Phenomenex, Torrance, CA, USA) with a flow rate of 0.3 mL/min. The mobile phase consisted of 10% methanol, adjusted to pH 2.8 with phosphoric acid (mobile phase A), and 100% methanol (mobile phase B) [32]. The gradient elution program was as follows: 52% B (0–3 min), 52–83% B (3–6 min), 83% B (6–10.5 min), and 52% B (10.5–13.5 min). The chromatographic data were processed using Agilent OpenLab CDS 3.2, and peak areas were analyzed for quantification.

### 2.6. Content Uniformity

The content uniformity of each drug layer was evaluated in accordance with USP <905> Uniformity of Dosage Units, which specifies testing of 10 individual units [33,34]. Ten bilayer tablets were randomly selected, and dissolved in 10 mL of 50% methanol, filtered through a 0.22 µm PVDF syringe filter, and diluted 50-fold. Drug concentrations were analyzed using UHPLC. The acceptance value (AV) was calculated using the following equation:

(1) $$AV=\left|M-\bar{X}\right|+ks,$$

where $\bar{X}$ is the mean of individual content values (%), s is the standard deviation of these values, and k is the acceptability constant (2.4). M is the reference value used for AV calculation. According to USP <905>, when the target content is 100% and $\bar{X}$ falls within 98.5–101.5%, M is set equal to $\bar{X}$. A formulation was considered compliant when AV ≤ 15.

### 2.7. Differential Scanning Calorimetry (DSC)

DSC was performed using a DSC 200 F3 Maia system (NETZSCH, Selb, Germany) to analyze the thermal properties of the samples. Approximately 2–3 mg of each sample (powdered drug, individual excipients, the physical mixture of LCD or MLS layer, and the 3D-printed LCD or MLS layer) was weighed into an aluminum pan and sealed. An empty pan was used as the reference. The samples were heated from 5 °C to 230 °C at a scanning rate of 10.0 °C/min under a nitrogen atmosphere at a flow rate of 60 mL/min.

### 2.8. Powder X-Ray Diffraction (PXRD)

The crystallinity of each powder, physical mixture, and 3D-printed sample was examined using a powder X-ray Diffraction (PXRD, D/MAX-2500V/PC, Rigaku, Tokyo, Japan). PXRD analysis was conducted using Cu-Kα radiation at 40 kV and 150 mA. The samples were scanned from 5° to 50° (2θ) at an increment of 0.02° and a speed of 1 s/scan. The presence of peaks was considered indicative of the crystalline form of the materials.

### 2.9. Scanning Electron Microscope (SEM)

The surface morphology of the bilayer tablets was assessed using field-emission scanning electron microscopy (FE-SEM, JSM-6700F, JEOL, Akishima, Japan). Samples were coated with platinum under vacuum for 150 s. Imaging was performed at various magnifications with a voltage of 5 kV.

### 2.10. In Vitro Dissolution Test

Dissolution test was conducted on 3D-printed bilayer tablets (*n* = 3) using a USP Apparatus II (LOGAN Instruments Corp., Somerset, NJ, USA) at a rotation speed of 100 rpm and a constant temperature of 37 ± 0.5 °C. For comparison, the corresponding free drug forms and a commercial reference product (Monterizine Cap.^®^, Hanmi Pharm Co., Ltd., Seoul, Republic of Korea) were evaluated under the same conditions.

The initial dissolution medium consisted of 750 mL of 0.1 N hydrochloric acid (pH 1.2) maintained at 37 ± 0.5 °C °C for 2 h. After the acidic stage, 250 mL of 0.2 M phosphate buffer was added to adjust the medium to pH 6.8, resulting in a total dissolution volume of 1000 mL. Sodium dodecyl sulfate (SDS, 0.5% *w*/*v*) was introduced only after the pH shift to the intestinal phase to improve the dissolution of MLS under intestinal-stage conditions [35,36]. The dissolution test was then continued for an additional 3 h under intestinal conditions. At predetermined time points (15, 30, 45, 60, 90, 120, 135, 150, 165, 180, 210, 240, and 300 min), 10 mL aliquots were withdrawn and immediately replaced with an equal volume of fresh medium to maintain a constant dissolution volume. Given that the nominal MLS dose was 10 mg and the final dissolution volume was 1000 mL, the dose/volume ratio was 0.01 mg/mL. On this basis, the pH 6.8 medium containing 0.5% SDS was considered to provide sink-like conditions for the MLS dose used in this study. All samples were filtered through a 0.22 µm PVDF syringe filter and analyzed by UHPLC at a detection wavelength of 220 nm.

### 2.11. Statistical Analysis

Data are expressed as mean ± standard deviation (SD). The number of replicates (*n*) is stated in the figure legends or methods, as appropriate. For comparisons among more than two groups, one-way analysis of variance (ANOVA) using GraphPad Prism 8 (GraphPad Software Inc., San Diego, CA, USA) followed by Tukey’s multiple comparisons test was used. In addition, dissolution profile comparison between the developed formulation and the reference product was assessed using the similarity factor (f_2_). A *p*-value less than 0.05 was considered statistically significant.

## 3. Results and Discussion

### 3.1. Optimization of Printing Parameters

To ensure the structural integrity and print quality of the bilayer tablets, the printing and moving speeds were carefully evaluated. Excessively slow or fast speeds resulted in disrupted printing lines and poor layer adhesion. Therefore, the available speed ranges in the software were systematically screened and suitable conditions were optimized for reliable printing. The optimized printing parameters, including printing speed, moving speed, infill pattern, filling density, and bed temperature, were standardized for both LCD and MLS layers. These conditions ensured consistent filament deposition, uniform infill, and effective interlayer integration, thereby achieving reproducible print quality. The printing of each layer required approximately 30 min, and the overall fabrication process for one bilayer tablet was approximately 1 h. Because the two layers were printed sequentially, this duration reflects the total fabrication time of the bilayer tablet rather than the continuous residence time of each formulation at the final set temperature in the heated barrel. The finalized settings are summarized in Table 1, which served as the basis for subsequent experiments and formulation evaluations.

### 3.2. Optimization of Polymer Combinations for 3D-Printed Tablets

**Figure 2:**
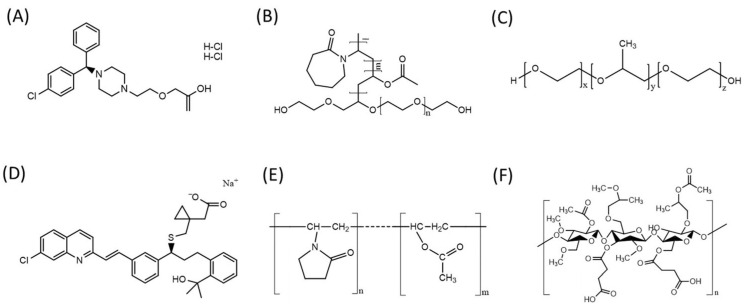
Chemical structures of polymers and active pharmaceutical ingredients (**A**) Levocetirizine dihydrochloride (LCD), (**B**) Montelukast sodium (MLS), (**C**) Soluplus^®^, (**D**) Kollidon^®^ VA 64 (PVPVA64), (**E**) Kolliphor^®^ P 188 (P188), (**F**) Hydroxypropyl methylcellulose acetate succinate (HPMC AS).

In this study, various polymer combinations were systematically evaluated for the development of 3D-printed bilayer tablets, considering not only theoretical formulation principles but also practical challenges encountered during printing and dissolution testing (Figure 2). For the immediate release layer containing LCD, Soluplus^®^ was initially selected as a single polymer because of its excellent thermal stability and suitability for HMPE. However, its high melt viscosity and brittle fracture properties resulted in filament breakage, making continuous printing difficult [37]. To mitigate this issue, poloxamer 188 (P188) was incorporated as a plasticizer, which improved extrusion stability and filament flexibility, thereby enabling stable printing (Supplementary Tables S1 and S2) [38,39]. Despite the improved printability, dissolution testing of these formulations revealed incomplete and delayed disintegration of the LCD layer, leading to suboptimal immediate release performance. To assess the effect of internal structural design, the infill density was systematically varied up to 50% (Supplementary Figure S1). Even though lower infill densities resulted in relatively faster release, none of the tested designs met the criteria for immediate release. The findings indicate that infill architecture influences release behavior, yet it is inadequate in itself to achieve immediate release, suggesting the necessity for further optimization of the polymer composition. To address this limitation, low-molecular-weight polyethylene oxide (Polyox™ N10, PEO) was introduced as a disintegration promoter [40]. PEO readily hydrates and swells upon contact with aqueous media, promoting matrix erosion and facilitating drug release. Ultimately, the combination of Soluplus^®^, P188, and PEO provided a suitable balance of printability, mechanical integrity, and rapid disintegration, and was selected as the optimized polymer system for fabricating the LCD immediate release layer (Table 2).

The delayed release layer containing MLS was formulated using the enteric polymer HPMC AS, which dissolves at pH values above 6.0, thus initiating drug release in the small intestine [41]. This design was consistent with the absorption characteristics of MLS, which is unstable in the stomach but absorbed predominantly in the intestine [27]. PEO enabled hydration-mediated erosion and modulated drug release upon intestinal exposure, while PVPVA64 promoted surface wetting and molecular dispersion of MLS within an amorphous glassy polymer matrix, thereby enhancing dissolution behavior. In addition, P188 further enhanced processability during extrusion. Considering that gastric emptying generally occurs within 2–4 h after oral administration, the HPMC AS-based delayed release design was intended to ensure that MLS would be released mainly after transit to the small intestine, thereby delaying systemic exposure compared with an immediate release formulation [42].

Taken together, the optimized bilayer design secured the processability of HMPE-based 3D printing while accommodating polymer combinations intended to provide immediate release of LCD and delayed release of MLS.

### 3.3. Mechanical Properties of Tablets

The size, mass, and hardness of the 3D-printed bilayer tablets were evaluated to assess the reproducibility and precision of the fabrication process. The tablets exhibited an average size of 12.1 ± 0.05 mm (width) × 7.5 ± 0.05 mm (length) × 2.8 ± 0.02 mm (height), with minimal variation observed among different batches (Table 3). The mean tablet weight was 216.1 ± 2.5 mg, indicating excellent reproducibility across printed units. This high level of dimensional and mass uniformity reflects the reliability of the HMPE 3D printing technique, in line with previous reports highlighting the precision of extrusion-based additive manufacturing for solid dosage forms [7,43].

The hardness of the tablets averaged 4.5 ± 0.1 kgf, which exceeds the minimum recommended hardness of approximately 4 kgf [44]. This result confirms that the tablets possess sufficient mechanical strength to withstand stresses during handling, storage, and packaging, comparable to values reported for other 3D-printed bilayer formulations [45]. Tablet hardness is a critical attribute, as it directly influences resistance to breakage and contributes to drug release performance. Factors such as excipient type and proportion, as well as tablet geometry and density, are known to impact mechanical strength. Thus, the obtained hardness values not only demonstrate the robustness of the present formulation but also support its suitability for practical pharmaceutical applications.

In addition to hardness testing, the friability of the printed bilayer tablets was evaluated to assess their mechanical robustness. The measured friability was 0.04%, which is well below the commonly accepted pharmacopeial limit of 1%, indicating sufficient resistance to mechanical stress during handling. Together with the hardness data, this result supports the mechanical stability of the printed tablets. No visible layer separation or structural failure was observed after friability testing, despite repeated tumbling and impact stress.

Optical microscopy and SEM observations further revealed continuous interfacial contact between the immediate-release and delayed-release layers without visible gaps or separation. In HMPE-based 3D printing, sequential deposition of adjacent layers may facilitate thermal interfacial fusion, which can help reduce the likelihood of delamination [46,47]. Consistent with this interpretation, no visible layer separation or structural failure was observed during handling, friability testing, or dissolution testing. These findings support adequate overall mechanical integrity and qualitative interfacial continuity of the printed bilayer tablets under the tested conditions.

### 3.4. Content Uniformity

The content uniformity of the bilayer tablets was evaluated according to USP <905>. Ten tablets were analyzed individually for both the LCD and MLS layers (Table 4). For the LCD layer, the drug content ranged from 97.45% to 102.98%, with a mean of 99.02%, an RSD of 1.63%, and an AV of 3.87. For the MLS layer, the drug content ranged from 97.59% to 103.30%, with a mean of 99.28%, an RSD of 1.82%, and an AV of 4.34. All values were well within the USP <905> acceptance limit (AV ≤ 15), confirming excellent content uniformity. The measured drug contents for both APIs were close to theoretical values and exhibited low variability (%RSD < 2%), indicating that the HMPE printing process did not adversely affect the assay of either API. These findings also provide supportive evidence that no substantial degradation occurred under the applied processing conditions. These results demonstrate the high reproducibility and accuracy of the formulation. The low variability across units further supports the accuracy of the 3D printing process and its reliability for manufacturing bilayer tablets with uniform dose distribution.

### 3.5. DSC

**Figure 3:**
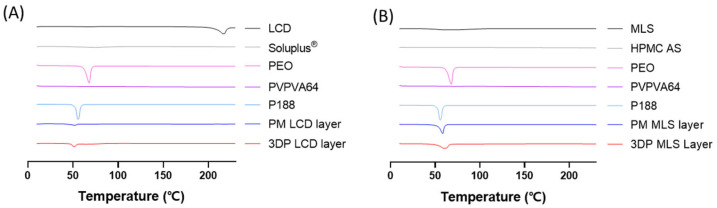
Differential scanning calorimetry (DSC) thermograms of the individual excipients, physical mixtures, and 3D-printed bilayer tablet formulations. (**A**) Thermograms of LCD, Soluplus^®^, PVPVA64, P188, physical mixture before 3D printing, and the 3D-printed LCD layer. (**B**) Thermograms of MLS, HPMC AS, PEO, PVPVA64, P188, physical mixture (PM) before 3D printing, and the 3D-printed MLS layer.

The thermal behavior of APIs, polymers, their physical mixtures, and 3D-printed tablets was evaluated using DSC (Figure 3). The DSC thermogram of LCD exhibited a sharp endothermic peak around 210 °C, corresponding to its melting point, confirming its crystalline nature [48] (Figure 3A). By contrast, no discernible endothermic peaks were observed for Soluplus^®^ and PVPVA64, indicating their amorphous character and concomitant thermal stability. P188 exhibited a characteristic melting peak at around 55 °C, consistent with its known thermal properties [49]. For the 3DP LCD layer, the endothermic LCD peak disappeared, indicating that the drug was converted into an amorphous or molecularly dispersed state within the polymeric matrix. The minor thermal event near 55 °C in the 3DP LCD layer was attributed to P188. The processing temperature (127 °C) was well below the melting point of LCD, indicating that the HMPE process was conducted under thermally compatible conditions for the drug. These results indicate that the solid-state conversion of LCD occurred as a consequence of the HMPE-based fabrication process rather than as a prerequisite for immediate-release performance. Accordingly, the observed amorphization was considered a processing outcome associated with polymer–drug interactions during extrusion and deposition, rather than a deliberate strategy for enhancing solubility or biopharmaceutical performance.

For MLS, the raw API used in this study already exhibited amorphous solid-state characteristics prior to printing. Specifically, the DSC thermogram of raw MLS did not show a distinct crystalline melting endotherm, which is consistent with an amorphous solid-state character (Figure 3B). The post-printing DSC profile of the MLS layer likewise did not show a crystallization-related thermal event. Therefore, the post-printing solid-state results support retention of the amorphous state of the raw MLS used in this study, rather than printing-induced amorphization. Consistent with this observation, previous thermogravimetric and thermal stress studies have shown that MLS remains stable below 300 °C and exhibits no significant degradation after exposure to 100 °C for up to 24 h, indicating a sufficiently wide thermal processing window for melt-based manufacturing [50]. Taken together, these findings suggest that HMPE-based 3D printing preserved the initial solid-state characteristics of the raw MLS used in this study under the applied fabrication conditions.

### 3.6. PXRD

**Figure 4:**
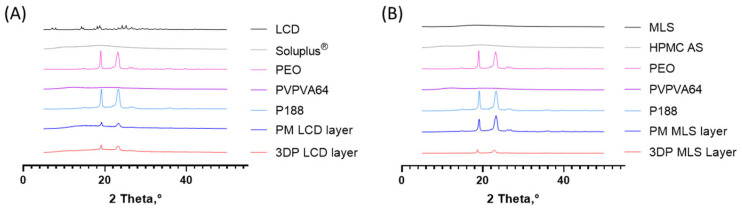
Powder X-ray diffraction (PXRD) patterns of the individual excipients, physical mixtures, and 3D-printed bilayer tablet formulations. (**A**) Diffraction patterns of LCD, Soluplus^®^, PVPVA64, P188, physical mixture (PM), and the 3D-printed LCD layer. (**B**) Diffraction patterns of MLS, HPMC AS, PEO, PVPVA64, P188, physical mixture (PM), and the 3D-printed MLS layer.

PXRD is a widely employed technique to investigate the crystalline or amorphous nature of pharmaceutical compounds and to monitor solid-state transformations induced during processing [51]. In this study, PXRD analysis was performed on APIs, excipients, and 3D-printed formulations to assess whether the HMPE process altered the solid-state characteristics (Figure 4).

As shown in Figure 4A, the diffractogram of pure LCD exhibited multiple sharp and intense peaks, particularly in the 2θ range of 10–30°, confirming its highly crystalline structure [52]. Conversely, Soluplus^®^ and PVPVA64 showed broad halo patterns without sharp peaks, consistent with amorphous materials, while P188 presented several well-defined diffraction peaks typical of crystalline substances (Figure 4A). After 3D printing, the characteristic crystalline peaks of LCD were not observed and were replaced by a broad halo, indicating complete amorphization of the drug during the HMPE process. This observation is consistent with the corresponding DSC data and suggests that the drug was molecularly dispersed within the polymeric matrix. The absence of crystalline diffraction peaks was interpreted as a solid-state transformation induced by the HMPE-based fabrication process, rather than as an indicator of enhanced biopharmaceutical performance. Given the high aqueous solubility of LCD, this amorphous state was regarded as a processing-related characteristic rather than a critical determinant of dissolution behavior.

For MLS, the raw API used in this study already exhibited amorphous solid-state characteristics prior to printing, as supported by the absence of distinct crystalline reflections in the DSC and PXRD results (Figure 3B and Figure 4B) [53]. Excipients incorporated in the MLS layer, such as HPMC AS and PVPVA64, also exhibited amorphous diffraction patterns, while PEO and P188 retained their crystalline signature (Figure 4B). After 3D printing, the PXRD pattern of the MLS layer remained consistent with the initial amorphous profile of the raw MLS, with no newly emerged distinct crystalline reflections attributable to recrystallized drug. Therefore, the post-printing solid-state results support retention of the amorphous state of the raw MLS used in this study, rather than printing-induced amorphization (Figure 4B). This retention of the initial amorphous state suggests that the thermal and mechanical conditions of the HMPE process did not induce detectable recrystallization of MLS under the applied processing conditions. Comparable results have been reported for other amorphous drugs processed by additive manufacturing, indicating that 3D printing can maintain drug integrity while enabling precise formulation design [8].

**Figure 5:**
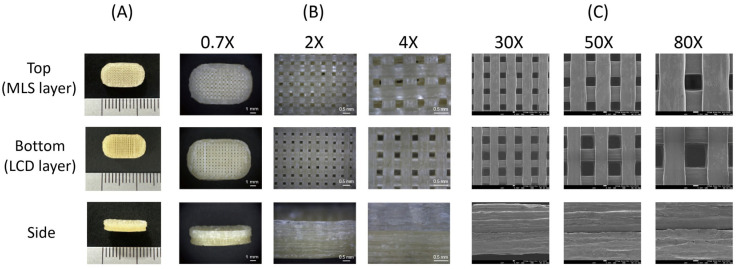
Surface morphology of the 3D-printed bilayer tablet. (**A**) Digital camera image, (**B**) zoom-stereo microscopy image, and (**C**) SEM image.

The thermal processing conditions used in this study were considered compatible with both APIs. MLS has been reported to remain stable below approximately 150 °C, while substantial degradation occurs only at higher temperatures, depending on the formulation system [54,55]. Given that the printing temperature for the MLS layer was 100 °C, significant thermal degradation during HMPE processing is unlikely. LCD likewise exhibits a reported melting range of approximately 218 °C, which is well above the printing temperature used for the LCD layer (127 °C) [48]. Importantly, the approximately 1 h required for fabrication represented the overall printing process for one bilayer tablet rather than the continuous residence time of each formulation at the final set temperature in the heated barrel. Because the two layers were printed sequentially and the barrel temperature was increased stepwise before deposition, the actual exposure time of each formulation at 100 °C (MLS) or 127 °C (LCD) was shorter than the total fabrication time. In addition, no visible darkening or browning was observed in the printed tablets (Figure 5), suggesting the absence of obvious thermal decomposition during processing. Consistent with these reports and observations, the measured drug contents of the printed tablets remained close to the theoretical values (99.02% for LCD and 99.28% for MLS; Table 4), and no significant change in drug content was observed after storage at room temperature for 4 weeks. These findings provide preliminary support for short-term chemical stability under ambient storage conditions. In addition, a recent study on MLS and LCD fixed-dose combination tablets demonstrated that the stability and compatibility of this API pair can be improved through appropriate formulation design [36]. In this context, the bilayer architecture used in the present study, which spatially separates the two APIs, may be advantageous in minimizing direct drug–drug contact within a single dosage form. However, the effects of longer-term storage on the solid-state characteristics and sequential dissolution behavior of the printed bilayer tablets remain to be evaluated.

### 3.7. Surface Morphology of 3D-Printed Bilayer Tablets

The surface and internal morphology of pharmaceutical tablets provide important insights into microstructural integrity, interlayer bonding, and potential influences on drug release [56,57]. To investigate these characteristics, bilayer tablets fabricated by HMPE-based 3D printing were characterized using a digital camera, zoom stereo microscopy, and SEM (Figure 5A, Figure 5B, and Figure 5C, respectively). The tablets were composed of a lower LCD layer and an upper MLS layer, both designed with a 50% infill lattice structure to balance mechanical strength and dissolution behavior. As shown in Figure 5, both the MLS (top) and LCD (bottom) layers exhibited clearly defined lattice networks with uniform pore distribution, while side-view images confirmed the bilayer arrangement and consistent stacking of filaments. Optical and SEM images revealed a highly regular lattice pattern with uniform filament spacing, demonstrating accurate layer deposition. High-magnification SEM showed distinct filament textures and the continuous interfacial bonding between the immediate-release and delayed-release layers without visible gaps or separation (Figure 5). These observations are consistent with the layer-by-layer deposition mechanism of HMPE-based 3D printing, in which sequential deposition of adjacent layers can promote thermal interfacial fusion and contribute to the structural integrity of the bilayer tablets.

This lattice structure is a key determinant of drug release performance. Both layers were printed under identical 3D printing settings; however, differences in formulation composition resulted in distinct microstructural features (Figure 5). The porous grid-like structure enables rapid solvent infiltration into the LCD layer, thereby promoting fast disintegration and immediate drug release. In contrast, the MLS layer showed a comparatively denser filament arrangement attributable to polymer composition, which contributed to matrix integrity and enabled sustained drug release, with the release kinetics modulated primarily by the enteric polymer composition. The use of a 50% infill density provided a reproducible framework in which formulation-dependent properties primarily governed the release behavior of each layer. Such structure–release relationships highlight the versatility of extrusion-based 3D printing, where both infill geometry and excipient selection can be modulated to achieve tailored drug release. Similar findings have been reported in other studies where internal architecture and material composition synergistically influenced dissolution performance [58].

### 3.8. In Vitro Dissolution of 3D-Printed Bilayer Tablets

**Figure 6:**
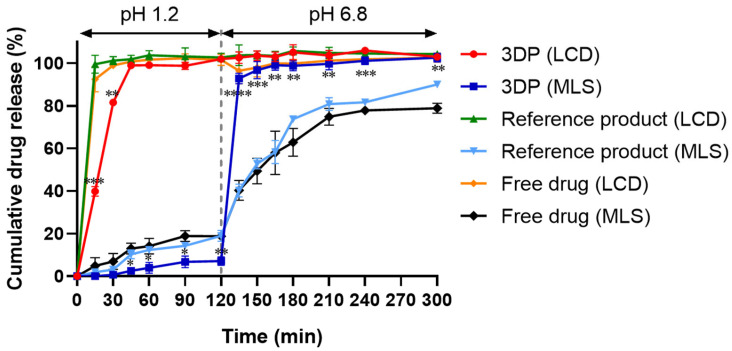
In vitro dissolution profiles of LCD and MLS from the 3D-printed bilayer tablet, compared with their respective free-drug forms and the commercial reference product (Monterizine Cap.^®^). The dissolution test was conducted under a pH-shift condition (0.1N HCl, pH 1.2 for 2 h, followed by phosphate buffer, pH 6.8 for 3 h). Data are expressed as mean ± SD (*n* = 3). * *p* < 0.05, ** *p* < 0.01, *** *p* < 0.001, **** *p* < 0.001 for the 3DP tablet versus the reference product at the corresponding sampling time point.

In vitro dissolution studies are essential to assess whether 3D-printed bilayer tablets achieve the desired release kinetics and to establish their performance from a formulation perspective [59]. The dissolution profiles of the 3D-printed bilayer tablets are presented in Figure 6. The dissolution study was performed sequentially in pH 1.2 medium for 2 h, followed by pH 6.8 medium for 3 h, simulating the transition from gastric to intestinal conditions as recommended by USP guidelines [60]. In the intestinal phase, 0.5% SDS was incorporated into the dissolution medium to maintain sink conditions for MLS, which exhibits limited aqueous solubility. Surfactant-containing dissolution media are commonly used to improve the dissolution of poorly water-soluble drugs and obtain reproducible dissolution profiles [61]. Similar approaches have been reported in dissolution studies of montelukast formulations and other poorly soluble drug products [62,63,64]. However, because surfactant-containing media may not fully reflect physiological intestinal conditions, the present dissolution data should be interpreted primarily as an in vitro comparison of formulation performance rather than as a direct predictor of in vivo release behavior. This experimental design enabled the assessment of immediate-release behavior under acidic conditions and enteric-protected release under intestinal conditions from a formulation standpoint.

The LCD exhibited rapid drug release in acidic medium (pH 1.2), with approximately 80% of the drug released within 30 min and complete release within 60 min. Although slightly slower than the reference product (Monterizine Cap.^®^) and free drug, which both reached near-complete release within 15 min, the observed release profile remained within generally accepted pharmacopeial expectations for immediate-release dosage forms defined in pharmacopeial dissolution testing (Figure 6) [65]. As shown in Supplementary Figure S1, PEO-free LCD layers with infill densities of 100%, 80%, and 50% released more slowly, with less than 80% dissolution at 30 min, highlighting the role of PEO as a disintegration-promoting component within the hydrophilic polymer matrix. Thus, the combination of Soluplus^®^, PVPVA64, P188, and PEO was considered suitable for achieving reproducible immediate-release behavior in the printed LCD layer. Comparable release profiles have been reported for other immediate-release formulations fabricated by extrusion-based 3D printing, supporting the applicability of this approach [66]. Statistical analysis at 15 and 30 min showed that LCD release from the 3D-printed bilayer tablet was significantly lower than that of the commercial reference product (*p* < 0.01), which is consistent with the slightly retarded initial release behavior of the polymer-based printed matrix.

To enable a criterion-based interpretation of delayed-release performance of the MLS layer, dissolution behavior was evaluated against stage-specific criteria commonly applied to delayed-release dosage forms and consistent with FDA guidance for modified-release oral products: not more than 10% drug release during 2 h at pH 1.2 and at least 80% drug release within 60 min after transition to pH 6.8 buffer [67]. At pH 1.2, MLS release from the 3D-printed bilayer tablet remained below 10% over 2 h, whereas the reference product (Monterizine Cap.^®^) and free MLS showed approximately 20% release during the same period. After the medium was shifted to pH 6.8, MLS release from the 3D-printed bilayer tablet increased rapidly, exceeded 80% within 15 min, and ultimately reached complete dissolution (Figure 6). Under these predefined criteria, the 3D-printed bilayer tablet satisfied both the acid-stage and buffer-stage requirements, thereby supporting the classification of the MLS-containing layer as a delayed-release component under the tested conditions. This biphasic release behavior—characterized by minimal release under acidic conditions followed by rapid release under intestinal conditions—confirms the functional separation of the two layers and reflects the pH-dependent solubility of the HPMC AS–based matrix. Statistical analysis further showed that MLS release from the 3D-printed bilayer tablet did not differ significantly from that of the reference product at 15 and 30 min (*p* > 0.05), whereas significant differences were observed from 45 min onward (*p* < 0.05), reflecting the distinct delayed-release behavior of the printed formulation.

To quantitatively compare the dissolution behavior with the reference product, the similarity factor (f_2_) was calculated according to regulatory guidance [68]. The calculated f_2_ values were 41.3 for LCD and 34.8 for MLS, both of which were below 50, indicating dissimilar dissolution profiles relative to the reference product for both APIs. These differences are consistent with the distinct release design of the HMPE-printed bilayer system, in which the polymer-based matrix structure was intended to provide immediate release of LCD and delayed release of MLS within a single dosage form, rather than to replicate the dissolution behavior of the reference product. Although the observed sequential release profile is consistent with the intended design of the bilayer system and may offer a formulation strategy for temporally separating the pharmacological actions of LCD and MLS, its chronotherapeutic and clinical relevance cannot be confirmed on the basis of the present in vitro dissolution data alone. Further pharmacokinetic and clinical studies are needed to establish an in vitro–in vivo correlation and to determine the therapeutic significance of this approach.

From a formulation and process perspective, these dissolution results demonstrate the successful integration of temporally separated release profiles within a single bilayer dosage form using HMPE-based 3D printing. The spatial separation of the IR and DR layers enabled distinct release behaviors without the need for post-printing coating or additional processing steps, in contrast to conventional bilayer or enteric-coated tablet manufacturing, which typically requires multiple unit operations to achieve comparable release characteristics [41]. Accordingly, this work highlights the practical applicability of extrusion-based additive manufacturing as a formulation platform for constructing multi-compartment oral dosage forms with distinct release behaviors.

## 4. Conclusions

This study described the fabrication of bilayer tablets containing MLS and LCD using HMPE-based 3D printing technology. The formulation was designed to provide complementary release profiles through immediate release of LCD and enteric-protected delayed release of MLS within a single dosage form. Based on comprehensive evaluations of physical characteristics, content uniformity, and in vitro dissolution, the printed tablets were found to be reproducible and technically viable from a formulation standpoint. The conversion of the active pharmaceutical ingredients into amorphous forms within the polymer matrix was confirmed by DSC and PXRD, while dissolution studies demonstrated distinct release phases consistent with the intended formulation design of each drug layer. Although the present design employed fixed doses, the inherent flexibility of 3D printing suggests opportunities for dose personalization in future applications. Beyond the present fixed-dose design, the digitally controlled HMPE platform offers opportunities for patient-specific dose adjustment and programmable release profiles, highlighting its potential in personalized oral combination therapy. Overall, the results demonstrate the feasibility of HMPE-based 3D printing for fabricating bilayer tablets integrating immediate-release LCD and delayed-release MLS, with reproducible dual-phase release and drug-specific release behavior within a single oral dosage form.

## Figures and Tables

**Table 1:** Optimized 3D printing slicer settings for bilayer tablet fabrication containing Levocetirizine Dihydrochloride (LCD) and Montelukast Sodium (MLS).

Slicer Settings	Set Values and Type	
	Levocetirizine Dihydrochloride (LCD)	Montelukast Sodium (MLS)
Printing Speed (mm/s)	2	2
Moving Speed (mm/s)	3	3
Infill pattern	Line	Line
Filling Density (%)	50	50
Bed Temperature (℃)	40	45
Printing temperature (℃)	127	100
Pneumatic pressure (kPa)	200	500

**Table 2:** Composition of 3D-printed bilayer tablets containing LCD and MLS.

Layer	LCD (%)	MLS (%)	Soluplus^®^ (%)	HPMC AS (%)	PEO (%)	PVPVA64 (%)	P188 (%)
LCD Layer	5	−	35	−	35	5	20
MLS Layer	−	9	−	10	63	13	5

(−): Not added. LCD; Levocetirizine dihydrochloride, MLS; Montelukast sodium.

**Table 3:** Physical characteristics of 3D-printed bilayer tablets containing LCD and MLS.

Tablets	Total Tablet Weight (mg)	LCD Layer Weight (mg)	MLS Layer Weight (mg)	Width (mm)	Length (mm)	Height (mm)	Hardness (kgf)
1	213.2	100.7	112.5	12.0	7.5	2.8	4.4
2	219.4	103.6	115.8	12.1	7.6	2.85	4.6
3	215.7	98.2	117.5	12.1	7.5	2.8	4.6
Mean ± SD	216.1 ± 2.5	100.8 ± 2.2	115.3 ± 2.1	12.1 ± 0.05	7.5 ± 0.05	2.8 ± 0.02	4.5 ± 0.1

LCD; Levocetirizine dihydrochloride, MLS; Montelukast sodium.

**Table 4:** Content uniformity and acceptance value (AV) of 3D-printed bilayer tablets containing LCD and MLS.

Parameters	LCD Layer	MLS Layer
Drug content (%)	98.10	99.06
	102.98	100.86
	98.75	97.63
	99.35	97.59
	99.31	97.69
	99.36	100.33
	97.68	98.83
	99.59	98.15
	97.66	99.31
	97.45	103.3
Mean (%)	99.02	99.28
%RSD	1.63	1.82
Acceptance value (AV)	3.87	4.34
Max. allowed AV (L1)	15	15

LCD; Levocetirizine dihydrochloride, MLS; Montelukast sodium.

## Data Availability

The original contributions presented in this study are included in the article and supplementary material. Further inquiries can be directed to the corresponding author.

## Supplementary Materials

The following supporting information can be downloaded at https://www.mdpi.com/article/10.3390/pharmaceutics18040444/s1: Figure S1: Drug Release profiles.; Table S1: Composition of the levocetirizine dihydrochloride (LCD) layer in bilayer tablets fabricated with different infill densities., Table S2: Physical properties of the LCD layer in bilayer tablets with different infill densities. References [65,69,70,71] are cited in the Supplementary Material.

## Abbreviations

The following abbreviations are used in this manuscript:

3DP	Three-dimensional printing
APIs	Active pharmaceutical ingredients
AV	Acceptance value
DR	Delayed release
DSC	Differential scanning calorimetry
FDM	Fused deposition modeling
HMPE	Hot-melt pneumatic extrusion
IR	Immediate release
LCD	Levocetirizine dihydrochloride
LTRAs	Leukotriene receptor antagonists
MLS	Montelukast sodium
PXRD	Powder X-ray diffraction
SEM	Scanning electron microscope
SD	Standard deviation
UHPLC	Ultra-high performance liquid chromatography

## References

[1] Kim Y.J., Choi Y.R., Kang J.H., Park Y.S., Kim D.W., Park C.W. (2024). Geometry-Driven Fabrication of Mini-Tablets via 3D Printing: Correlating Release Kinetics with Polyhedral Shapes. Pharmaceutics.

[2] Kim J.H., Kim K., Jin H.-E. (2022). Three-dimensional printing for oral pharmaceutical dosage forms. J. Pharm. Investig..

[3] Majrashi M.A.A., Yahya E.B., Mushtaq R.Y., Abdul Khalil H.P.S., Rizg W.Y., Alissa M., Alkharobi H., Badr M.Y., Hosny K.M. (2024). Revolutionizing drug delivery: Exploring the impact of advanced 3D printing technologies on polymer-based systems. J. Drug Deliv. Sci. Technol..

[4] Karalia D., Siamidi A., Karalis V., Vlachou M. (2021). 3D-Printed Oral Dosage Forms: Mechanical Properties, Computational Approaches and Applications. Pharmaceutics.

[5] Wang N., Shi H., Yang S. (2022). 3D printed oral solid dosage form: Modified release and improved solubility. J. Control Release.

[6] Serrano D.R., Kara A., Yuste I., Luciano F.C., Ongoren B., Anaya B.J., Molina G., Diez L., Ramirez B.I., Ramirez I.O. (2023). 3D Printing Technologies in Personalized Medicine, Nanomedicines, and Biopharmaceuticals. Pharmaceutics.

[7] Cho H.W., Baek S.H., Lee B.J., Jin H.E. (2020). Orodispersible Polymer Films with the Poorly Water-Soluble Drug, Olanzapine: Hot-Melt Pneumatic Extrusion for Single-Process 3D Printing. Pharmaceutics.

[8] Kim S.J., Lee J.C., Ko J.Y., Lee S.H., Kim N.A., Jeong S.H. (2021). 3D-printed tablets using a single-step hot-melt pneumatic process for poorly soluble drugs. Int. J. Pharm..

[9] Deshkar S., Rathi M., Zambad S., Gandhi K. (2021). Hot Melt Extrusion and its Application in 3D Printing of Pharmaceuticals. Curr. Drug Deliv..

[10] Bandari S., Nyavanandi D., Dumpa N., Repka M.A. (2021). Coupling hot melt extrusion and fused deposition modeling: Critical properties for successful performance. Adv. Drug Deliv. Rev..

[11] Kumari J., Pandey S., Jangde K.K., Kumar P.V., Mishra D.K. (2024). Evolution, Integration, and Challenges of 3D Printing in Pharmaceutical Applications: A Comprehensive Review. Bioprinting.

[12] Shamji M.H., Durham S.R. (2017). Mechanisms of allergen immunotherapy for inhaled allergens and predictive biomarkers. J. Allergy Clin. Immunol..

[13] Acevedo-Prado A., Seoane-Pillado T., Lopez-Silvarrey-Varela A., Salgado F.J., Cruz M.J., Faraldo-Garcia A., Nieto-Fontarigo J.J., Pertega-Diaz S., Sanchez-Lastres J., San-Jose-Gonzalez M.A. (2022). Association of rhinitis with asthma prevalence and severity. Sci. Rep..

[14] Savoure M., Bousquet J., Jaakkola J.J.K., Jaakkola M.S., Jacquemin B., Nadif R. (2022). Worldwide prevalence of rhinitis in adults: A review of definitions and temporal evolution. Clin. Transl. Allergy.

[15] Nappi E., Paoletti G., Malvezzi L., Ferri S., Racca F., Messina M.R., Puggioni F., Heffler E., Canonica G.W. (2022). Comorbid allergic rhinitis and asthma: Important clinical considerations. Expert. Rev. Clin. Immunol..

[16] Horak F., Zieglmayer P.U., Zieglmayer R., Kavina A., Lemell P. (2005). Levocetirizine has a longer duration of action on improving total nasal symptoms score than fexofenadine after single administration. Br. J. Clin. Pharmacol..

[17] Simons F.E.R., Simons K.J. (2011). Histamine and H1-antihistamines: Celebrating a century of progress. J. Allergy Clin. Immunol..

[18] Meltzer E.O., Philip G., Weinstein S.F., LaForce C.F., Malice M.P., Dass S.B., Santanello N.C., Reiss T.F. (2005). Montelukast effectively treats the nighttime impact of seasonal allergic rhinitis. Am. J. Rhinol..

[19] Dykewicz M.S., Wallace D.V., Amrol D.J., Baroody F.M., Bernstein J.A., Craig T.J., Dinakar C., Ellis A.K., Finegold I., Golden D.B.K. (2020). Rhinitis 2020: A practice parameter update. J Allergy Clin Immunol..

[20] Kim M.K., Lee S.Y., Park H.S., Yoon H.J., Kim S.H., Cho Y.J., Yoo K.H., Lee S.K., Kim H.K., Park J.W. (2018). A Randomized, Multicenter, Double-blind, Phase III Study to Evaluate the Efficacy on Allergic Rhinitis and Safety of a Combination Therapy of Montelukast and Levocetirizine in Patients With Asthma and Allergic Rhinitis. Clin. Ther..

[21] Kim C.K., Hwang Y., Song D.J., Yu J., Sohn M.H., Park Y.M., Lim D.H., Ahn K., Rha Y.H. (2024). Efficacy and Safety of Montelukast+Levocetirizine Combination Therapy Compared to Montelukast Monotherapy for Allergic Rhinitis in Children. Allergy Asthma Immunol. Res..

[22] Shao M., Sun J., Zheng Q. (2025). Efficacy and safety of montelukast-levocetirizine combination therapy in combined allergic rhinitis and asthma syndrome: A systematic review and meta-analysis. J. Asthma.

[23] Cobanoglu B., Toskala E., Ural A., Cingi C. (2013). Role of leukotriene antagonists and antihistamines in the treatment of allergic rhinitis. Curr. Allergy Asthma Rep..

[24] Zhao L., Yang J., Liu T., Cao H., Liang Y., Wang B. (2024). Comparison of clinical research trends and hotspots in allergic rhinitis and asthma from 2013 to 2023 based on bibliometric analysis. Heliyon.

[25] Shen C., Chen F., Wang H., Zhang X., Li G., Wen Z. (2020). Individualized treatment for allergic rhinitis based on key nasal clinical manifestations combined with histamine and leukotriene D4 levels. Braz. J. Otorhinolaryngol..

[26] Jordan A., Toennesen L.L., Eklof J., Sivapalan P., Meteran H., Bonnelykke K., Ulrik C.S., Staehr Jensen J.U. (2023). Psychiatric Adverse Effects of Montelukast-A Nationwide Cohort Study. J. Allergy Clin. Immunol. Pract..

[27] Al Omari M.M., Zoubi R.M., Hasan E.I., Khader T.Z., Badwan A.A. (2007). Effect of light and heat on the stability of montelukast in solution and in its solid state. J. Pharm. Biomed. Anal..

[28] Loh Y.Y., Enose A.A., Garg V. (2022). Enteric-Coated Polymers Past and Present-A Review. Drug Deliv. Letters.

[29] Simons F.E., Simons K.J. (2005). Levocetirizine: Pharmacokinetics and pharmacodynamics in children age 6 to 11 years. J. Allergy Clin. Immunol..

[30] Lee J., Song C., Noh I., Song S., Rhee Y.-S. (2020). Hot-melt 3D extrusion for the fabrication of customizable modified-release solid dosage forms. Pharmaceutics.

[31] Krueger L., Cao Y., Zheng Z., Ward J., Miles J.A., Popat A. (2023). 3D printing tablets for high-precision dose titration of caffeine. Int. J. Pharm..

[32] Hasan N., Siddiqui F., Afridi N., Chaiharn M., Khan S., Abrar M. (2012). A New Acetonitrile-Free, Cost-Effective, Simple and Validated Rp-HPLC Method for Determination of Montelukast Sodium in Bulk, Tablets and Liquid Dosage Forms. J. Anal. Bioanal. Tech..

[33] Mitra B., Thool P., Meruva S., Aycinena J.A., Li J., Patel J., Patel K., Agarwal A., Karki S., Bowen W. (2020). Decoding the small size challenges of mini-tablets for enhanced dose flexibility and micro-dosing. Int. J. Pharm..

[34] The United States Pharmacopeial Convention (2011). General Chapter. <905> Uniformity of Dosage Unite, The United States Pharmacopeia and The National Formulary. https://www.usp.org/sites/default/files/usp/document/harmonization/gen-method/q0304_stage_6_monograph_25_feb_2011.pdf.

[35] Zaid A.N., Natour S., Qaddomi A., Abu Ghoush A. (2013). Formulation and in vitro and in vivo evaluation of film-coated montelukast sodium tablets using Opadry(R) yellow 20A82938 on an industrial scale. Drug Des. Dev. Ther..

[36] Yun T.H., Kim M.J., Lee J.G., Bang K.H., Kim K.S. (2024). Enhanced Stability and Compatibility of Montelukast and Levocetirizine in a Fixed-Dose Combination Monolayer Tablet. Pharmaceutics.

[37] Monteil M., Sanchez-Ballester N.M., Devoisselle J.M., Begu S., Soulairol I. (2024). Regulations on excipients used in 3D printing of pediatric oral forms. Int. J. Pharm..

[38] Desai D., Sandhu H., Shah N., Malick W., Zia H., Phuapradit W., Vaka S.R.K. (2018). Selection of Solid-State Plasticizers as Processing Aids for Hot-Melt Extrusion. J. Pharm. Sci..

[39] Szafraniec-Szczęsny J., Antosik-Rogóż A., Kurek M., Gawlak K., Górska A., Peralta S., Knapik-Kowalczuk J., Kramarczyk D., Paluch M., Jachowicz R. (2021). How does the addition of Kollidon^®^ VA64 inhibit the recrystallization and improve ezetimibe dissolution from amorphous solid dispersions?. Pharmaceutics.

[40] Gultekin H.E., Tort S., Acarturk F. (2019). An Effective Technology for the Development of Immediate Release Solid Dosage Forms Containing Low-Dose Drug: Fused Deposition Modeling 3D Printing. Pharm. Res..

[41] Goyanes A., Fina F., Martorana A., Sedough D., Gaisford S., Basit A.W. (2017). Development of modified release 3D printed tablets (printlets) with pharmaceutical excipients using additive manufacturing. Int. J. Pharm..

[42] Abell T.L., Camilleri M., Donohoe K., Hasler W.L., Lin H.C., Maurer A.H., McCallum R.W., Nowak T., Nusynowitz M.L., Parkman H.P. (2008). Consensus recommendations for gastric emptying scintigraphy: A joint report of the American Neurogastroenterology and Motility Society and the Society of Nuclear Medicine. J. Nucl. Med. Technol..

[43] Dumpa N., Butreddy A., Wang H., Komanduri N., Bandari S., Repka M.A. (2021). 3D printing in personalized drug delivery: An overview of hot-melt extrusion-based fused deposition modeling. Int. J. Pharm..

[44] Deshpande R.D., Gowda D., Mahammed N., Maramwar D.N. (2011). Bi-layer tablets-An emerging trend: A review. Int. J. Pharm. Sci. Res..

[45] Yang H.S., Kim D.W. (2023). Fabrication of Gastro-Floating Famotidine Tablets: Hydroxypropyl Methylcellulose-Based Semisolid Extrusion 3D Printing. Pharmaceutics.

[46] Abebe A., Akseli I., Sprockel O., Kottala N., Cuitiño A.M. (2014). Review of bilayer tablet technology. Int. J. Pharm..

[47] Barocio E., Brenken B., Favaloro A., Pipes R.B. (2022). Interlayer fusion bonding of semi-crystalline polymer composites in extrusion deposition additive manufacturing. Compos. Sci. Technol..

[48] Labib G.S. (2015). Novel levocetirizine HCl tablets with enhanced palatability: Synergistic effect of combining taste modifiers and effervescence technique. Drug Des. Dev. Ther..

[49] Hu X.Y., Lou H., Hageman M.J. (2018). Preparation of lapatinib ditosylate solid dispersions using solvent rotary evaporation and hot melt extrusion for solubility and dissolution enhancement. Int. J. Pharm..

[50] Azizoglu E., Ozer O. (2020). Fabrication of Montelukast sodium loaded filaments and 3D printing transdermal patches onto packaging material. Int. J. Pharm..

[51] Thakral S., Terban M.W., Thakral N.K., Suryanarayanan R. (2016). Recent advances in the characterization of amorphous pharmaceuticals by X-ray diffractometry. Adv. Drug Deliv. Rev..

[52] Karothu D.P., Alhaddad Z., Gob C.R., Schurmann C.J., Bucker R., Naumov P. (2023). The Elusive Structure of Levocetirizine Dihydrochloride Determined by Electron Diffraction. Angew. Chem. Int. Ed. Engl..

[53] Barbosa J.S., Lysenko K., Paz F.A.A., Braga S.S. (2021). Inclusion of Montelukast in y-Cyclodextrin: Presenting a Mechanochemical Route to Improve Drug Stability and Solubility. Proceedings.

[54] Dolatabadi A.K., Mokhtari J., Talebian N. (2023). Silica xerogel carrier as encapsulating material for the in-vitro controlled release of montelukast. Inorg. Chem. Commun..

[55] Lee H.R., Park H.J., Park J.S., Park D.W., Ho M.J., Kim D.Y., Lee H.C., Kim E.J., Song W.H., Park J.S. (2021). Montelukast microsuspension with hypromellose for improved stability and oral absorption. Int. J. Biol. Macromol..

[56] Fanous M., Bitar M., Gold S., Sobczuk A., Hirsch S., Ogorka J., Imanidis G. (2021). Development of immediate release 3D-printed dosage forms for a poorly water-soluble drug by fused deposition modeling: Study of morphology, solid state and dissolution. Int. J. Pharm..

[57] Oh D.J., Shin S.Y., Lim C.Y., Chae Y.B., Yang J.H., Hwang S.J. (2025). Development of a stable fixed-dose combination of Montelukast Sodium and Levocetirizine Dihydrochloride using multi-layering API coating technology. Int. J. Pharm..

[58] Thakkar R., Pillai A.R., Zhang J., Zhang Y., Kulkarni V., Maniruzzaman M. (2020). Novel on-demand 3-dimensional (3-D) printed tablets using fill density as an effective release-controlling tool. Polymers.

[59] Shuklinova O., Wyszogrodzka-Gaweł G., Baran E., Lisowski B., Wiśniowska B., Dorożyński P., Kulinowski P., Polak S. (2024). Can 3D Printed Tablets Be Bioequivalent and How to Test It: A PBPK Model Based Virtual Bioequivalence Study for Ropinirole Modified Release Tablets. Pharmaceutics.

[60] Lee J.H., Park C., Weon K.Y., Kang C.Y., Lee B.J., Park J.B. (2021). Improved Bioavailability of Poorly Water-Soluble Drug by Targeting Increased Absorption through Solubility Enhancement and Precipitation Inhibition. Pharmaceuticals.

[61] Zhao Y., Feng Y., Ren X., Hou Y., Wang F., Wang R., Guo M. (2024). Preparation and properties characterization of composite modified starch-based orodispersible films. J. Appl. Pharm. Sci..

[62] Janugade B., Patil S., Patil S., Lade P. (2009). Formulation and evaluation of press-coated montelukast sodium tablets for pulsatile drug delivery system. Int. J. Chem. Tech. Res..

[63] Hadi M.A., Lokeswara Babu V., Pal N., Srinivasa Rao A. (2012). Formulation and evaluation of Sustained release Matrix tablets of Montelukast Sodium. Int. J. Pharm..

[64] Natarajan R., Jananipriya S., Jayaseelan S., Ganesan V. (2022). Design, optimization, formulation and evaluation of sustained release matrix tablets of montelukast sodium. World J. Pharm. Res..

[65] Charoo N.A., Abdallah D.B., Ahmed D.T., Abrahamsson B., Cristofoletti R., Langguth P., Mehta M., Parr A., Polli J.E., Shah V.P. (2023). Biowaiver Monograph for Immediate-Release Solid Oral Dosage Forms: Levocetirizine Dihydrochloride. J. Pharm. Sci..

[66] Ashokbhai M.K., Sanjay L.R., Roy S., Velayutham R., Kaity S. (2024). Mechanochemical Evaluation of a Hot Melt Extruded Ready-to-Print Etoricoxib Macrofilament as Printing Ink for Additive Manufacturing. Ind. Eng. Chem. Res..

[67] US Food and Drug Administration, Center for Drug Evaluation and Research (1997). SUPAC-MR: Modified Release Solid Oral Dosage Forms Scale-Up and Post Approval Changes: Chemistry, Manufacturing, and Controls; In Vitro Dissolution Testing, and In Vivo Bioequivalence Documentation. https://www.fda.gov/regulatory-information/search-fda-guidance-documents/supac-mr-modified-release-solid-oral-dosage-forms-scale-and-postapproval-changes-chemistry.

[68] Lee T.J., Kim D., Kim J.C., Ro S.W., Na D.H. (2023). Formulation development and pharmacokinetic evaluation of enteric-coated dexrabeprazole tablets. J. Pharm. Investig..

[69] FDA (2018). Dissolution Testing and Acceptance Criteria for Immediate-Release Solid Oral Dosage form Drug Products Containing High Solubility Drug Substances. https://www.fda.gov/media/92988/download.

[70] Cantin O., Siepmann F., Danede F., Willart J.F., Karrout Y., Siepmann J. (2016). PEO hot melt extrudates for controlled drug delivery: Importance of the molecular weight. J. Drug Deliv. Sci. Technol..

[71] Deshmukh J., Sanil K., Cherif A., Ashour E.A. (2025). Development of fenofibrate solid dispersion via hot melt extrusion and 3D printing technologies. Pharm. Dev. Technol..

