# Supplementary material for: Hot-Melt Pneumatic Extrusion-Based 3D-Printed Bilayer Tablets Enabling Sequential Release of Levocetirizine and Montelukast
Source: Pharmaceutics. 2026 Apr 3;18(4):444. doi: 10.3390/pharmaceutics18040444 (PMC13118815; doi:10.3390/pharmaceutics18040444)
Supplement: Supplementary file 1 [file pharmaceutics-18-00444-s001.zip › pharmaceutics-4172870-supplementary.pdf]

Supporting Information

Article

Hot-Melt Pneumatic Extrusion-Based 3D-Printed Bilayer Tablets Enabling Sequential Release of Levocetirizine and Montelukast

Ga-Ram Kim [1], Ji-Young Cho [1], Seung-Wuk Lee [2] and Hyo-Eon Jin [1,3,*]

[1] Department of Biohealth Regulatory Science, Graduate School of Ajou University, Suwon 16499, Republic of Korea; garam0431@ajou.ac.kr (G.-R.K.); jyoung919@ajou.ac.kr (J.-Y.C.)
[2] Department of Bioengineering, University of California, Berkeley, CA 94720, USA; leesw@berkeley.edu
[3] Department of Pharmacy, Ajou University, Suwon 16499, Republic of Korea
* Correspondence: hjin@ajou.ac.kr; Tel.: +82-31-219-3466

EXPERIMENTAL SECTION

Materials and instruments

Levocetirizine dihydrochloride (LCD, BLD Pharmatech Ltd, Shanghai, China) and montelukast sodium (MLS, Angene Chemical, London, UK) were used as active pharmaceutical ingredients (APIs). Excipients included Soluplus® (BASF Pharma, New Jersey, USA), POLYOX WSR N10 LEO (Colorcon Ltd, Dartford, UK), Kollidon® VA 64 (PVPVA64, BASF Pharma, New Jersey, USA), and Kolliphor® P188 (poloxamer 188, BASF Pharma, New Jersey, USA). All other chemicals were of analytical grade and were used as received.

The bilayer tablet was designed in Fusion 360 software (Autodesk, Inc., San Rafael, CA, USA) and printed using a hot-melt pneumatic extrusion (HMPE) 3D printer (ROKIT INVIVO 4D2, ROKIT INVIVO Corp., Seoul, Korea). Three-dimensional model files were then converted to G-code using NewCreatorK software (ROKIT INVIVO Corp., Seoul, Korea). Dissolution testing employed USP Apparatus II (LOGAN Instruments, Somerset, NJ, USA), and samples were analyzed by UHPLC using a 1290 Infinity II system (Agilent Technologies, Santa Clara, CA, USA).

Preparation of the LCD immediate-release layer and fabrication of 3D-printed bilayer tablets

To prepare the LCD immediate-release (IR) layer, the active pharmaceutical ingredient (API) and excipients were weighed according to the compositions listed in Table S1, for a total weight of 3 g. The powders were mixed thoroughly in a mortar and pestle for 10 min to obtain a homogeneous blend mixture, which was then transferred to the printing barrel and heated at 127°C for 30 min. The molten mixture was extruded at 200 kPa through a 0.3 mm nozzle

using a HMPE 3D printer. The LCD layers were printed at infill densities of 100%, 80%, and 50% to assess the effect of internal porosity on drug release, producing layers with dimensions of 12 mm × 7.5 mm × 1.35 mm. The montelukast sodium (MLS) layer was then deposited onto the printed LCD layer using the same HMPE system to construct the bilayer tablet, resulting in final dimensions of 12 mm × 7.5 mm × 2.7 mm.

***In Vitro* Dissolution test of 3D-printed bilayer tablets**

Bilayer tablets containing LCD layers printed at infill densities of 100%, 80%, or 50% were tested (n = 3 per condition) to evaluate the effect of internal porosity on drug release. The tests were performed using USP Apparatus II (LOGAN Instruments Corp., Somerset, NJ, USA) at 100 rpm and 37 ± 0.5°C. The initial dissolution medium was 750 mL of pH 1.2 buffer, in which tablets were tested for 2 hrs. Subsequently, 250 mL of 0.2 M sodium phosphate solution was added to the vessel, and the pH was adjusted to 6.8 with 1N hydrochloric acid (HCl). Sodium dodecyl sulfate (SDS) at 0.5% w/v was included in the pH 6.8 medium to enhance drug solubility. Dissolution was continued for an additional 3 hrs (total 5 hrs). At predetermined time points (15, 30, 45, 60, 90, 120, 135, 150, 165, 180, 210, 240, and 300 min), a 10 mL aliquot was withdrawn and immediately replaced with an equal volume of fresh medium to maintain sink conditions. Samples were filtered through 0.22 μm PVDF syringe filters and analyzed by UHPLC (Agilent Technologies, Santa Clara, CA, USA) at a detection wavelength of 220 nm.

Optimization of the LCD layer to achieve immediate drug release in bilayer tablet

The LCD layer composed of Soluplus® 70%, PVPVA64 5%, and Kolliphor® P188 (P188) 20% was first printed at an infill density of 100% as a single-layer structure (12 mm × 7.5 mm × 1.35 mm). This monolayer formulation exhibited complete drug release within 30 min, satisfying the immediate-release (IR) criterion (data not shown). However, when the LCD layer was incorporated into the bilayer configuration with the MLS layer, the cumulative release failed to meet the IR specification of ≥ 80% within 30 min (USP <711>; ICH Q6A). This reduction in release rate suggested that the bilayer geometry or interlayer interaction impeded drug diffusion from the LCD layer. To recover the desired IR performance, the LCD formulation was further optimized. The initial Soluplus®-based formulations (Soluplus® 70%, PVP VA64 5%, and P188 20%) were printed at infill densities of 100%, 80%, and 50% to investigate the influence of internal porosity on drug release (Table S1 and S2). However, none of these formulations achieved ≥ 80% LCD release within 30 min, even at 50% infill, indicating that the dissolution rate was limited by the matrix composition rather than the geometric structure (Figure S1). These results indicate that the initial Soluplus®-based LCD formulations did not meet the pharmacopeial immediate-release specification when incorporated into a bilayer configuration. (USP <711>; ICH Q6A) (Charoo et al. 2023).

To address this limitation, the polymer composition of the LCD layer was modified by incorporating polyethylene oxide (PEO) as a co-polymer with Soluplus® to enhance hydrophilicity, swelling, and disintegration (Deshmukh et al. 2025). The final optimized formulation consisted of Soluplus® 35%, PEO 35%, PVPVA64 5%, and P188 20% (Table 2). The redesigned LCD layer was printed at an infill density of 50%, providing sufficient porosity and mechanical stability. As a result, the modified formulation achieved ≥ 80% cumulative

LCD release within 30 min (Figure S1). The enhanced release behavior was attributed to formulation-driven changes in matrix wettability, hydration, and erosion associated with the incorporation of PEO. The swelling and erosion characteristics of PEO are known to depend on its molecular weight, with high-molecular-weight PEO exhibiting pronounced swelling and slow erosion, whereas low-molecular-weight PEO hydrates and erodes more rapidly (Cantin et al. 2016). Accordingly, the inclusion of low-molecular-weight PEO (N10 grade) was considered effective in promoting matrix disintegration under dissolution conditions. This formulation was therefore selected for subsequent fabrication of bilayer tablets to ensure reproducible immediate-release performance based on pharmacopeial criteria (Figure S1).

Table S1. Composition of the levocetirizine dihydrochloride (LCD) layer in bilayer tablets fabricated with different infill densities.

**Infill (%)**	**LCD (%)**	**Soluplus® (%)**	**PVPVA64 (%)**	**P188 (%)**
100	5	70	5	20
80	5	70	5	20
50	5	70	5	20

LCD; Levocetirizine dihydrochloride

Table S2. Physical properties of the LCD layer in bilayer tablets with different infill densities.

Infill (%)	Weight (mg)	Width (mm)	Length (mm)	Height (mm)	Hardness (kgf)
100	217.2 ± 0.93	12.1 ± 0.08	7.5 ± 0.05	2.8 ± 0.02	5.75 ± 0.18
80	218.9 ± 0.29	12.1 ± 0.05	7.5 ± 0.05	2.8 ± 0.02	5.22 ± 0.41
50	214.7 ± 3.33	12.0 ± 0.05	7.5 ± 0.02	2.7 ± 0.02	4.39 ± 0.28

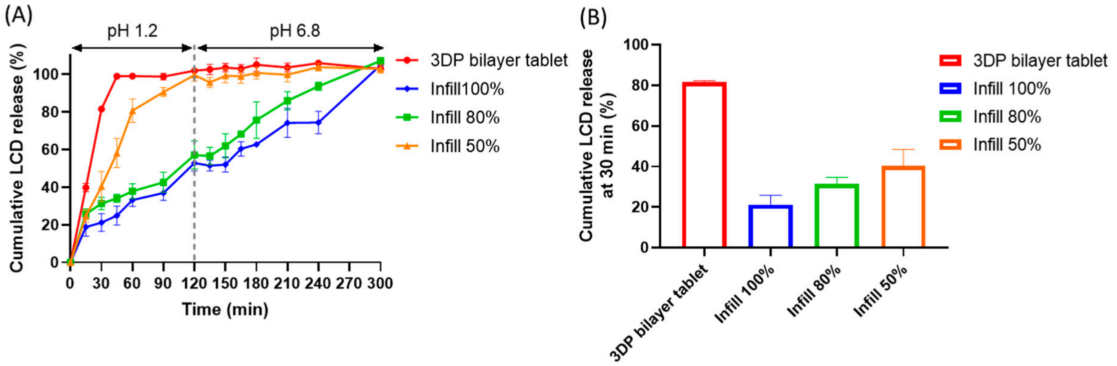

Figure S1. Drug Release profiles. (A) Drug release profiles of LCD from 3D-printed bilayer tablets fabricated with different infill densities of LCD layer (100%, 80%, and 50%) compared with the final 3DP bilayer tablet. (B) Cumulative LCD release at 30 min from bilayer tablets, each containing an LCD layer printed at different infill densities (100%, 80%, and 50%). Data are represented as mean ± SD (*n* = 3).

References

FDA. Dissolution testing and acceptance criteria for immediate-release solid oral dosage form drug products containing high solubility drug substances. 2018. Available at: https://www.fda.gov/media/92988/download. (Accessed 7 September 2022).

Cantin, O, Florence Siepmann, Florence Danede, JF Willart, Y Karrout, and J Siepmann. 2016. 'PEO hot melt extrudates for controlled drug delivery: Importance of the molecular weight', *Journal of Drug Delivery Science and Technology*, 36: 130-40.

Charoo, N. A., D. B. Abdallah, D. T. Ahmed, B. Abrahamsson, R. Cristofoletti, P. Langguth, M. Mehta, A. Parr, J. E. Polli, V. P. Shah, A. Kambayashi, and J. Dressman. 2023. 'Biowaiver Monograph for Immediate-Release Solid Oral Dosage Forms: Levocetirizine Dihydrochloride', *J Pharm Sci*, 112: 893-903.

Deshmukh, J., K. Sanil, A. Cherif, and E. A. Ashour. 2025. 'Development of fenofibrate solid dispersion via hot melt extrusion and 3D printing technologies', *Pharm Dev Technol*, 30: 852-62.
